# Nilotinib-Induced Immune-Mediated Liver Injury: Corticosteroid as a Possible Therapeutic Option

**DOI:** 10.3389/fonc.2020.01160

**Published:** 2020-07-10

**Authors:** Hyun Yang, Pil Soo Sung, Eun Sun Jung, Sungwoo Cho, Si Hyun Bae, Dong-Wook Kim

**Affiliations:** ^1^Division of Gastroenterology and Hepatology, Department of Internal Medicine, College of Medicine, Eunpyeong St. Mary's Hospital, The Catholic University of Korea, Seoul, South Korea; ^2^Division of Gastroenterology and Hepatology, Department of Internal Medicine, College of Medicine, Seoul St. Mary's Hospital, The Catholic University of Korea, Seoul, South Korea; ^3^The Catholic University Liver Research Center, College of Medicine, The Catholic University of Korea, Seoul, South Korea; ^4^Department of Hospital Pathology, College of Medicine, Eunpyeong St. Mary's Hospital, The Catholic University of Korea, Seoul, South Korea; ^5^Division of Hematology, Department of Internal Medicine, College of Medicine, Seoul St. Mary's Hospital, The Catholic University of Korea, Seoul, South Korea

**Keywords:** hepatotoxicity, drug induced liver injury, nilotinib, chronic myeloid leukemia, corticosteroid

## Abstract

**Introduction:** Nilotinib is a BCR-ABL tyrosine kinase inhibitor approved for chronic myeloid leukemia. We present a case of severe immune-mediated liver injury by nilotinib treatment.

**Case report:** A 59-year-old woman was referred to the liver clinic because of elevated liver enzyme levels. One year prior, she was diagnosed as having chronic myeloid leukemia and treated with nilotinib therapy. The level of aspartate aminotransferase and alanine aminotransferase were 578 IU/L and 499 IU/L, respectively. Percutaneous needle liver biopsy showed extensive centrilobular infiltration of immune cells and destruction of the lobular architecture with minimal inflammation in the portal triad. Immunohistochemical staining showed that many CD8^+^ T cells and CD56^+^ cells infiltrated the site of inflammation. Multicolor fluorescence-activated cell-sorting analysis revealed that a considerable number of intrahepatic CD8+ T cells showed an activated phenotype compared with the healthy control. She was diagnosed with nilotinib-induced, immune-mediated liver injury. Prednisolone treatment (30 mg daily) was started and caused rapid normalization of liver enzyme levels.

**Conclusion:** Nilotinib can cause immune-mediated liver injury. The use of corticosteroid can be treatment option in immune-mediated liver injury.

## Background

Nilotinib is a BCR-ABL tyrosine kinase inhibitor with superior potency to imatinib. Elevations in serum aminotransferases may occur during nilotinib therapy, but elevations greater than 5 times the upper limit of normal is a rare event. Until now, the precise mechanism of the liver injury by nilotinib is largely unknown. Here, we report a patient who developed severe immune-mediated liver injury by nilotinib treatment. Written informed consent was obtained from the patient described in this case report.

## Case Summary

A 59-year-old woman was referred to the liver clinic because of elevated liver enzyme levels.

**Figure 1:**
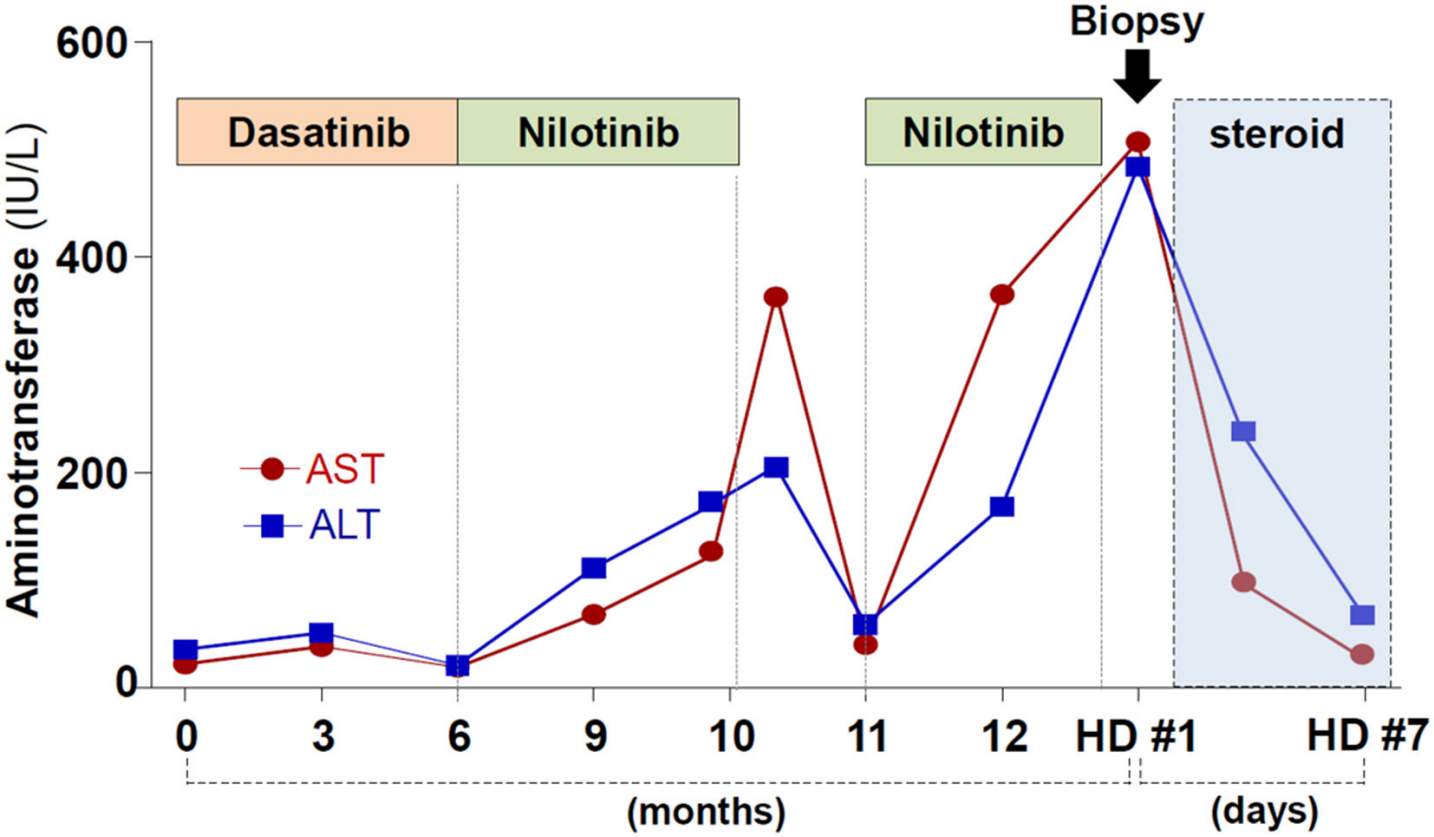
Summary of the patient progress after the diagnosis with chronic myeloid leukemia.

One year prior, she was diagnosed as having chronic myeloid leukemia. The patient was carrying the e14a2 (b3a2) BCR-ABL1 transcript. Initially, dasatinib treatment was started, but the drug caused intractable pleural effusion. Dasatinib was changed to nilotinib (300 mg, twice a day). Four months of nilotinib treatment resulted in the complete hematologic response in bone marrow examination, although it caused elevation of serum aminotransferase levels. Nilotinib was temporarily stopped, and liver enzymes became normalized. However, retrial of nilotinib after one month of drug holiday caused liver enzymes to rise more rapidly (Figure 1). She did not drink alcohol and denied taking any medications except for nilotinib.

The hemoglobin level was 13.8 g/dL; leukocyte count, 4,320/μL; and platelet count, 123,000/μL. There was no eosinophilia. Her aspartate aminotransferase level was 578 IU/L; alanine aminotransferase level, 499 IU/L; alkaline phosphatase level, 110 IU/L; gamma-glutamyl transferase level, 180 U/L; and total bilirubin level, 1.51 mg/dL. The prothrombin time was not prolonged. Test results for HBsAg, anti-HAV IgM antibody, anti-HCV antibody, and anti-HEV IgM antibody were negative. Serological results for human immunodeficiency virus, Epstein-Barr virus, and cytomegalovirus were all negative. The serum immunoglobulin G level was 1,383 mg/dL. The antinuclear antibody titer was 1:320. Results for anti-liver/kidney microsomal, anti-mitochondrial, anti-smooth muscle, and anti-neutrophil cytoplasmic antibodies were negative. Ultrasonography revealed a mildly increased peripheral echogenicity, without evidence of chronic liver disease or biliary obstruction.

**Figure 2:**
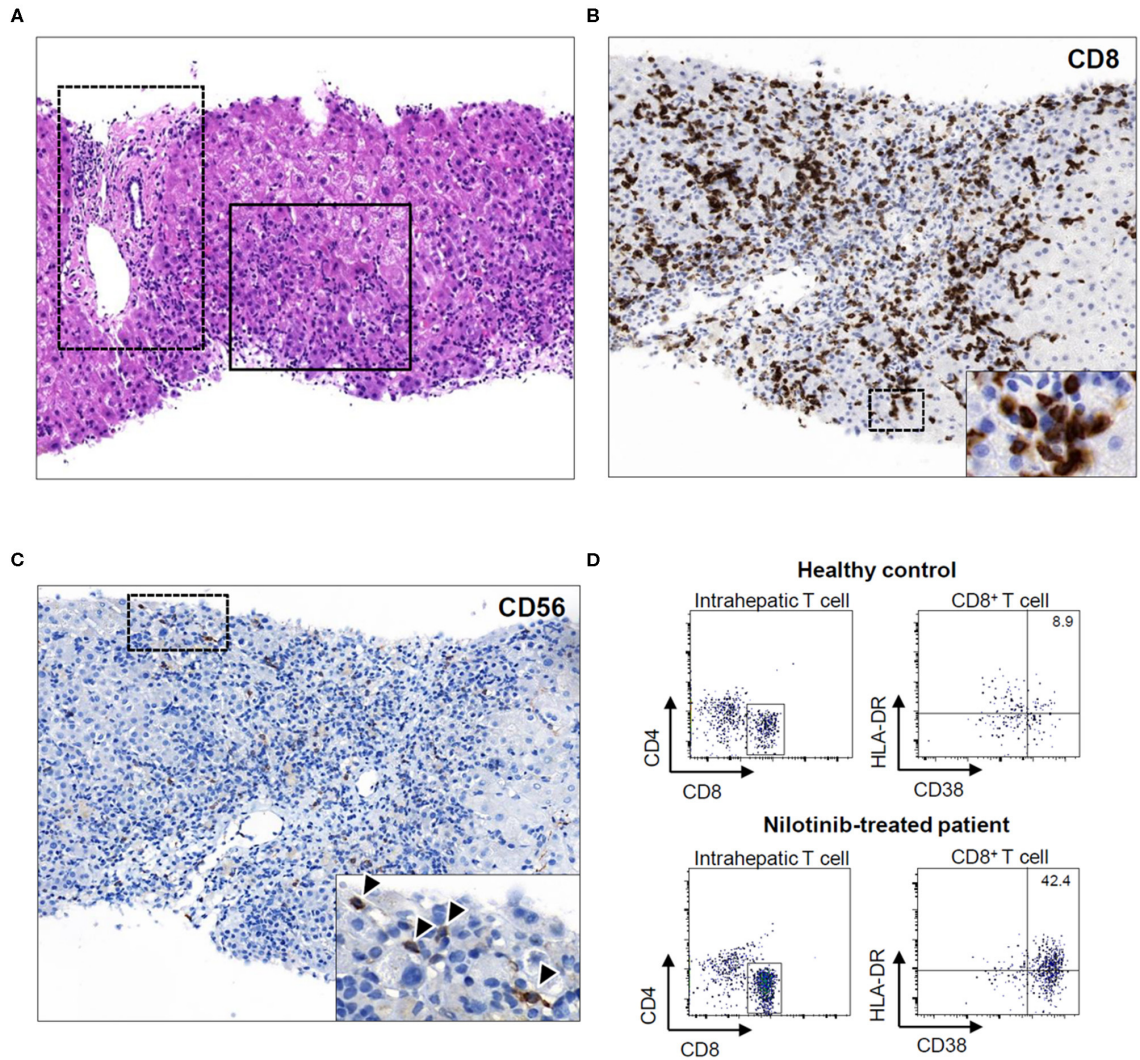
**(A)** Hematoxylin and eosin-stained section (Original magnification X100). Solid-lined square focuses on the centrilobular architecture and dot-lined square depicts the portal triad. **(B)** CD8 stain (Original magnification X200). **(C)** CD56 stain (Original magnification X200). **(D)** CD38 and HLA-DR staining results of intrahepatic CD8^+^ T cells.

Percutaneous needle liver biopsy on the second day showed extensive centrilobular infiltration of immune cells and destruction of the lobular architecture (solid-lined square) with minimal inflammation in the portal triad (dot-lined square; Figure 2A). Portal or perisinusoidal fibrosis was not detected. Immunohistochemical staining for CD8 showed that numerous CD8^+^ T cells were in contact with dying hepatocytes (Figure 2B). CD56 staining demonstrated that many CD56^+^ cells, including natural killer (NK) cells, also infiltrated the site of inflammation (arrowheads; Figure 2C). Multicolor fluorescence-activated cell-sorting analysis using liver biopsy specimens revealed that a considerable number of intrahepatic CD8^+^ T cells in the patient (42.4%) showed an activated phenotype (CD38^+^ HLA-DR^+^) compared with those in the healthy liver (8.9%; Figure 2D).

She was diagnosed with nilotinib-induced, immune-mediated liver injury. Prednisolone treatment (30 mg daily) was started and caused rapid normalization of liver enzyme levels (Figure 1). Prednisolone was gradually tapered and stopped after 14 days of use. After cessation of the corticosteroid therapy, the liver enzyme level remained normalized.

## Discussion

Nilotinib is a drug approved for the treatment of chronic myeloid leukemia by the Food and Drug Administration in 2007. The phase 3 randomized trial showed that nilotinib was superior to imatinib in major molecular and cytogenetic response (1). The incidence of grade 3 or 4 elevation of liver enzymes caused by nilotinib was reported 1 to 4% (2). Belopolsky et al. reported a case of the patient with nilotinib-induced grade 4 hepatotoxicity, which was normalized after the cessation of nilotinib (3). In that report, the liver biopsy showed acute hepatitis with severe portal and lobular inflammation, although the exact types of infiltrating immune cells or their activation status were not studied (3). There have also been several case reports of imatinib-induced liver injury, which did not identify the actual “killer cells” in the liver.

The mechanism of drug-induced liver injury (DILI) is heterogeneous and includes direct hepatotoxicity, immune-mediated liver injury, and mitochondria-selective toxicity (4). Several drugs have been reported to cause “immune-mediated DILI.” Sometimes, these patients may be indistinguishable from those with autoimmune hepatitis on the basis of their histological and clinical presentations (5, 6). The latency period between drug exposure and the onset of the disease can vary from 1 week to more than 3 months in the cases of immune-mediated DILI (5, 6). The absence of advanced fibrosis or cirrhosis, together with the features of hypersensitivity, a latency period to liver injury from drug exposures, and published reports on the suspected drug may support the diagnosis of immune-mediated DILI (7). Neoantigens are formed during the metabolism of the causative agent, and presented by antigen-presenting cells and hepatocytes. After priming, neoantigen-specific cytotoxic CD8^+^ cells may be expanded in the liver or migrate from circulation (7). Analysis of liver-infiltrating lymphocytes from the patients with immune-mediated DILI demonstrated that the number of activated CD8^+^ T-cells correlates with the degree of liver injury (7, 8). Serum aminotransferase levels > 3-fold upper normal limit (UNL) and total serum bilirubin level > 2-fold UNL may require corticosteroid therapy (5). Sustained biochemical resolution following the withdrawal of corticosteroid confirms the diagnosis of immune-mediated DILI rather than autoimmune hepatitis.

In our patient, liver biopsy showed extensive centrilobular infiltration of CD8^+^ T cells and NK cells, so-called “killer” cells, which suggests immune-mediated liver injury. The findings that most of the intrahepatic CD8^+^ T cells showed an activated phenotype (CD38^+^ HLA-DR^+^) are consistent with those of a recent study that demonstrated that activated CD8^+^ T cells of acute hepatitis A patients exert innate-like cytotoxicity, which is associated with liver injury (9). During acute hepatitis A, a considerable number of CD8^+^ T cells are activated in a T cell receptor-independent manner, and this phenomenon may also occur in the inflamed liver with activated killer cells triggered by certain drugs (10). The limitations of this report are as follows: First, there was the possibility of other causes of liver injury in our patient. Second, we are reporting only one case. Future studies with more cases are needed to validate of this report. In summary, our case shows immune-mediated liver injury by nilotinib and the possibility of therapeutic use of corticosteroids in immune-mediated liver injury.

## Data Availability Statement

The original contributions presented in the study are included in the article/supplementary material, further inquiries can be directed to the corresponding author/s.

## Ethics Statement

The studies involving human participants were reviewed and approved by The Institutional Review Board of The Catholic University of Korea. The patients/participants provided their written informed consent to participate in this study.

## Author Contributions

HY and PS: study concept and design, acquisition of data, analysis and interpretation of data, drafting of the manuscript, and critical revision of the manuscript for important intellectual content. SC: acquisition of data. EJ, SB, and D-WK: critical revision of the manuscript for important intellectual content. All authors contributed to the article and approved the submitted version.

## Conflict of Interest

The authors declare that the research was conducted in the absence of any commercial or financial relationships that could be construed as a potential conflict of interest.

## Abbreviations

HAV: hepatitis A virus
HBsAg: hepatitis B surface antigen
HCV: hepatitis C virus
HEV: hepatitis E virus
NK: natural killer
DILI: drug-induced liver injury.
